# Supplementary material for: Structure of the Staphylococcus aureus bacteriophage 80α neck shows the interactions between DNA, tail completion protein and tape measure protein
Source: bioRxiv. 2024 Dec 11:2024.12.10.627806. Preprint. [Version 1] doi: 10.1101/2024.12.10.627806 (PMC11661146; doi:10.1101/2024.12.10.627806)
Supplement: Supplement 1 — Figure S1. Fourier Shell Correlation (FSC) curves from cryoSPARC for the C6 (A) and C1 (B) reconstructions. Figure S2. Comparison of 80α neck proteins with the equivalent proteins from other phages. (A) HTCP: 80α gp49 (blue), SPP1 gp15 (red), Lambda gpW (purple), HK97 gp6 (yellow), JBD30 gp41 (green), GTA Rcc01688 (tan). (B) HTJP: 80α gp50 (green), SPP1 gp16 (red), lambda gpFII (blue), GTA Rcc01689 (tan). (C) TrP: 80α gp52 (pink), lambda gpU (blue), GTA Rcc01690 (tan). [file media-1.pdf]

SUPPLEMENTARY FIGURE LEGENDS

Figure S1. Fourier Shell Correlation (FSC) curves from cryoSPARC for the C6 (A) and C1 (B) reconstructions.

Figure S2. Comparison of 80α neck proteins with the equivalent proteins from other phages. (A) HTCP: 80α gp49 (blue), SPP1 gp15 (red), Lambda gpW (purple), HK97 gp6 (yellow), JBD30 gp41 (green), GTA Rcc01688 (tan). (B) HTJP: 80α gp50 (green), SPP1 gp16 (red), lambda gpFII (blue), GTA Rcc01689 (tan). (C) TrP: 80α gp52 (pink), lambda gpU (blue), GTA Rcc01690 (tan).

SUPPLEMENTARY FIGURES

Figure S1

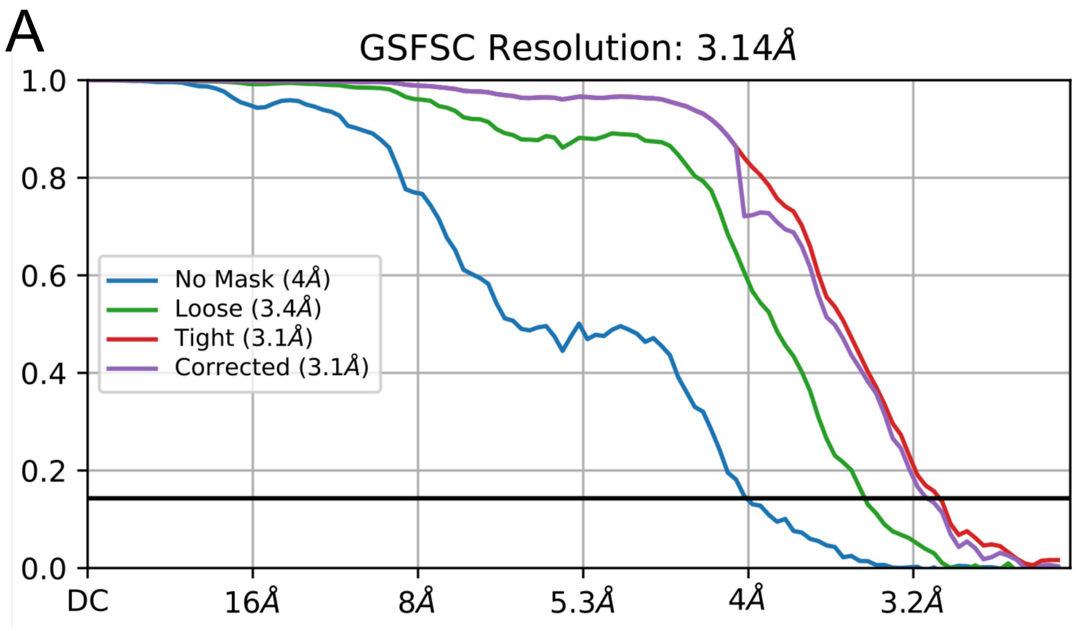

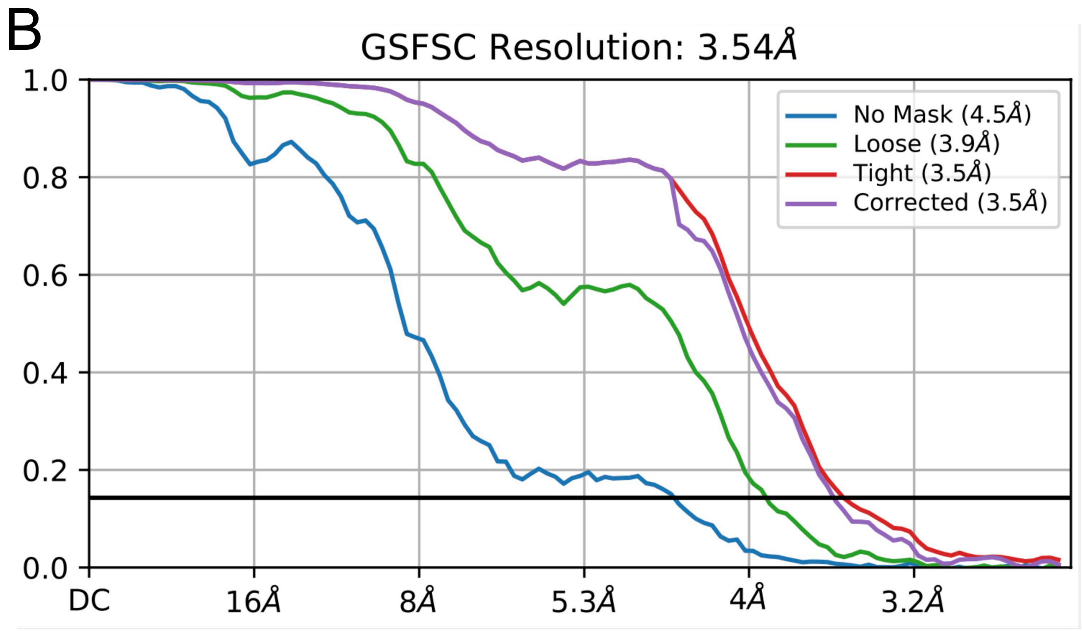

Figure S2

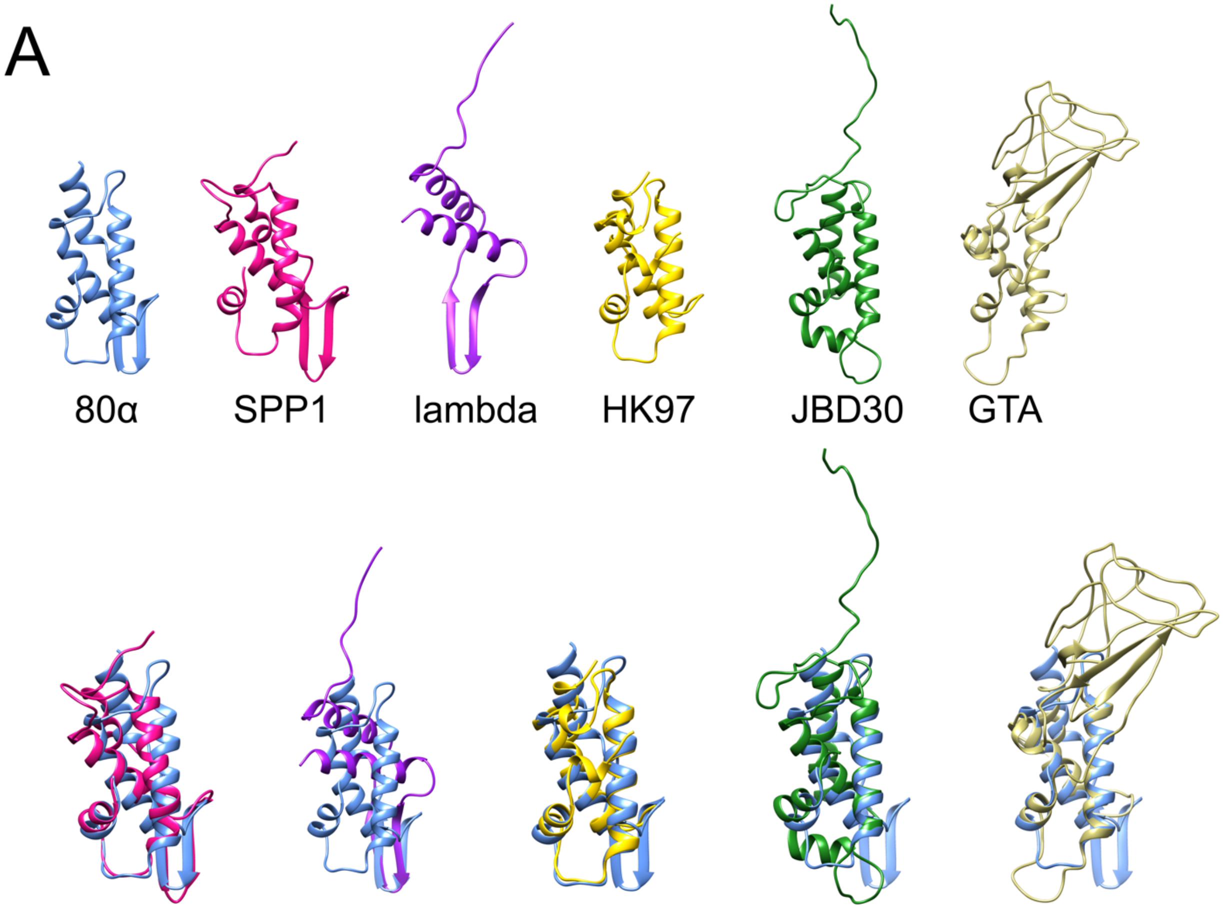

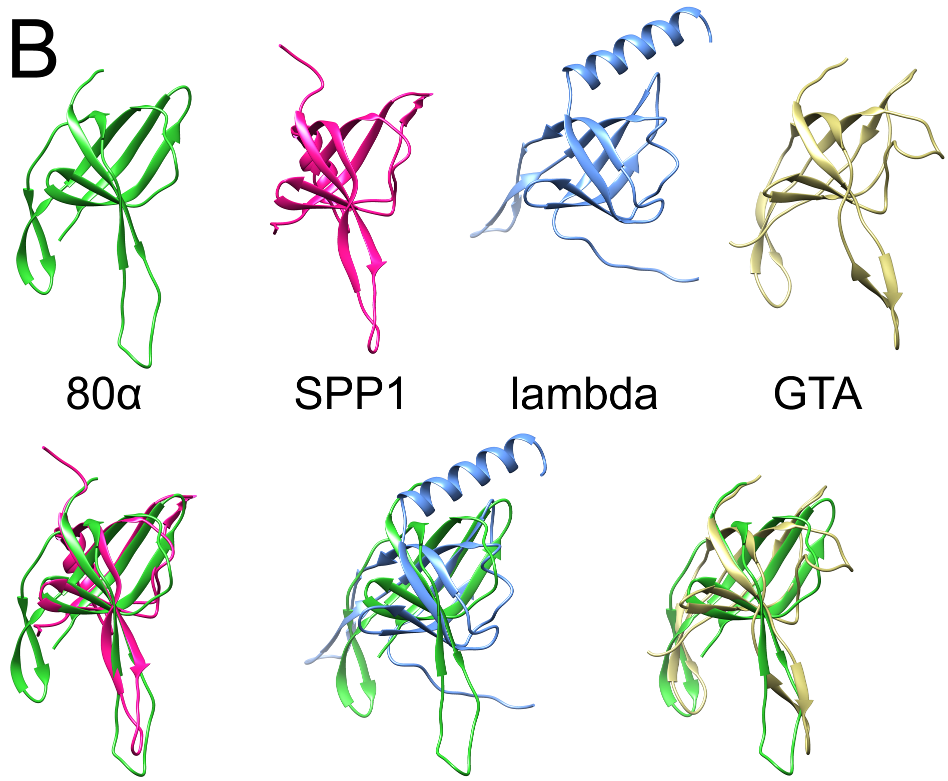
B
80α
SPP1
lambda
GTA

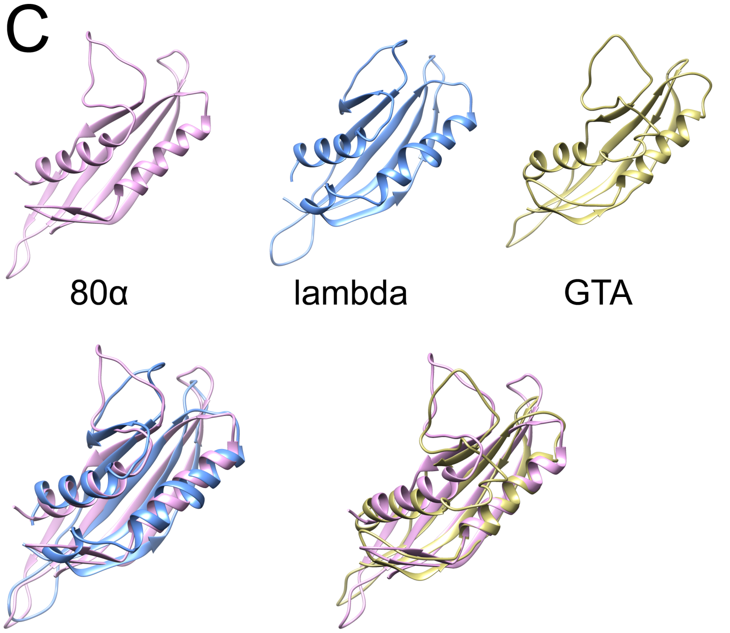
C
80α
lambda
GTA

SUPPLEMENTARY TABLES

Supplementary Table S1. (A) Data collection and processing parameters and statistics.

**Structure**	**Neck C6**	**Neck C1**
Microscope	FEI Titan Krios G1	
Camera	Gatan K3	
Energy filter	Gatan Quantum GIF	
Image collection software	Leginon	
Slit width (eV)	20	
Voltage (kV)	300	
Micrographs collected	2,796	
Defocus range (µm)	0.7–2.0	
Total exposure ($e^-/Å^2$)	35.26	
Frames per movie	38	
Pixel size (Å/pix)	1.33	
Reconstruction software	RELION-4.0; CryoSPARC v4.2	
Final particles	59,457	35,724
Symmetry imposed	C6	C1
Map resolution (Å, $FSC_{0.143}$)	3.1	3.5
EMDB Accession	EMD-XXXX	EMD-YYYY

Supplementary Table S1. (B) Model building parameters and statistics.

Structure	Neck C6	Neck C1
Refinement resolution (Å)	3.1	3.5
Model composition:		
Chains	6	42
Atoms	4,822	31,584
Hydrogens	0	0
Protein residues	593	3,779
Nucleotides	0	40
Waters	0	0
Ligands	0	0
Bonds (RMSD):		
Length (Å) (# > 4σ)	0.006 (0)	0.006 (0)
Angles (°) (# > 4σ)	1.230 (0)	1.152 (0)
MolProbity score	0.69	0.88
Clash score	0.00	0.52
Ramachandran plot (%):		
Favored	69.90	96.73
Allowed	3.10	3.27
Outliers	0.00	0.00
Rama-Z score (RMSD), N:		
Whole	-0.45 (0.33), N=581	-0.69 (0.13), N=3,699
Helix	-1.46 (0.35), N=153	-1.34 (0.13), N=1,055
Sheet	0.41 (0.37), N=185	0.24 (0.15), N=1,212
Loop	0.16 (0.40), N=243	0.03 (0.16), N=1,432
Rotamer outliers (%)	0.0	0.0
C_β outliers (%)	0.0	0.0
Peptide plane (%):		
Cis proline/general	12.5/0.0	12.2/0.0
Twisted proline/general	0.0/0.0	0.0/0.0
CaBLAM outliers (%)	0.35	0.50
Model fit vs. Map:		
$FSC_{0.5}$ ($FSC_{0.143}$)	3.4 (3.1)	3.7 (3.5)
CC_{volume}	0.80	0.83
CC_{mask}	0.86	0.83
Accession (PDB)	XXXX	YYYY

Supplementary Table S2. Root-mean-square deviations (RMSD) of equivalent Cα atoms between 80α/SaPI1 neck proteins and the equivalent proteins from other bacteriophages.

**80α/SaPI1**		**vs.**	**SPP1**	**PDB ID: 7Z4W**			
Protein	Gene product	# res	gp	Total¶	RMSD	Pruned§	RMSD
HTCP	gp49	110	gp15	93	4.84	52	2.26
HTJP	gp50	100	gp16	89	6.99	49	1.57

**80α/SaPI1**		**vs.**	**Lambda**	**PDB ID: 8K38**			
	Gene product	# res	gp	total	RMSD	pruned	RMSD
HTCP	gp49	110	gpW	49	6.59	23	2.34

**80α/SaPI1**		**vs.**	**Lambda**	**PDB ID: 8K37**			
Protein	Gene product	# res	gp	total	RMSD	pruned	RMSD
HTJP	gp50	100	gpFII	84	14.43	25	2.28
TrP	gp52	127	gpU	114	5.42	48	2.34

**80α/SaPI1**		**vs.**	**HK97**	**PDB ID: 3JVO**			
Protein	Gene product	# res	gp	total	RMSD	pruned	RMSD
HTCP	gp49	110	gp6	85	3.61	64	1.66

**80α/SaPI1**		**vs.**	**JBD30**	**PDB ID: 8RKB**			
Protein	Gene product	# res	gp	total	RMSD	pruned	RMSD
HTCP	gp49	110	gp41	81	5.21	55	1.78

**80α/SaPI1**		**vs.**	**GTA**	**PDB ID: 6TE9**			
Protein	Gene product	# res	gp	total	RMSD	pruned	RMSD
HTCP	gp49	110	Rcc01688	58	23.31	28	1.05
HTJP	gp50	100	Rcc01689	91	7.25	53	1.61
TrP	gp52	127	Rcc01690	110	10.86	49	1.62

¶Total=total number of residue pairs compared. The RMSD value to the right is between this number of residue pairs.

§Pruned=number of residue pairs compared after pruning to remove all pairs with an RMSD>3.5Å, and the corresponding RMSD value. Aligned in UCSF Chimera using the BLOSUM-62 matrix and 50% weight on secondary structure vs. sequence.
